# Angular spectrum method for curvilinear arrays: Theory and application to Fourier beamforming

**DOI:** 10.1121/10.0010536

**Published:** 2022-05-13

**Authors:** Rehman Ali, Jeremy Dahl

**Affiliations:** 1 Department of Electrical Engineering, Stanford University, Stanford, California 94305, USA; 2 Department of Radiology, Stanford University School of Medicine, Palo Alto, California 94304, USA rali8@alumni.stanford.edu, jjdahl@stanford.edu

## Abstract

Fourier beamforming techniques for medical ultrasound imaging have largely been limited to linear transducer arrays. This work extends the angular spectrum method to curvilinear arrays and demonstrates a migration-based Fourier beamforming technique that has implications for sound speed estimation and distributed aberration correction for abdominal imaging applications. When compared to Field II simulations, the proposed angular spectrum method simulates the pressure field from a focused transmission to within 3.7% normalized root mean square error. The resulting Fourier beamforming technique is then compared to virtual source synthetic aperture using *in vivo* abdominal imaging examples where resolution and imaging quality improvements are observed.

## 1. Introduction

The angular spectrum method is commonly used in medical ultrasound to simulate pressure fields induced by a linear or planar transducer array by propagating the plane wave decomposition of the wavefield from one depth plane to the next (Schafer and Lewin, 1989). Recently, the angular spectrum method was used to perform pulse-echo ultrasound imaging (Ali, 2021) with linear arrays based on a seismic imaging technique known as shot-profile migration (Schleicher *et al.*, 2008). Shot-profile migration consists of propagating and correlating transmit and receive wavefields to produce an image. Because shot-profile migration was performed using the angular spectrum method, all computations were performed in the Fourier domain, resulting in a new Fourier-domain beamformer.

The application of this new Fourier beamforming technique to pulse-echo ultrasound (Ali, 2021) yielded significant imaging improvements over conventional virtual-source synthetic aperture (Bae and Jeong, 2000) and REFoCUS (Ali *et al.*, 2020; Bottenus, 2018) with focused transmissions, and f-k Stolt's migration (Garcia *et al.*, 2013) with plane wave transmissions. However, the application of this and previous Fourier beamforming techniques to medical ultrasound imaging has been limited to linear transducer arrays (Ali, 2021; Cheng and Lu, 2006; Garcia *et al.*, 2013; Moghimirad *et al.*, 2016; Schwab and Schmitz, 2018). In the case of shot-profile migration, this limitation stems from the fact that the angular spectrum method requires a Cartesian coordinate system.

Although the angular spectrum method has previously been used to simulate wavefields from curved transducer arrays (Ilyina *et al.*, 2017; Vyas and Christensen, 2011), these approaches either require conversion to Cartesian grid by approximating a curved source as a series of planar sources (Ilyina *et al.*, 2017) or use a very specific spherical geometry unsuitable for applications involving curvilinear arrays (Vyas and Christensen, 2011). The goal of this work is to present a polar form of the angular spectrum method that enables Fourier beamforming for abdominal applications involving curvilinear probes.

## 2. Theory of the angular spectrum method in polar coordinates

The two-dimensional wave equation can be written as

$$\frac{\partial^{2}p(x,z,t)}{\partial x^{2}}+\frac{\partial^{2}p(x,z,t)}{\partial z^{2}}=\frac{1}{c^{2}}\frac{\partial^{2}p(x,z,t)}{\partial t^{2}},$$ (1)

where p(x,z,t) is the pressure at location (*x*, *z*) and time *t*, and *c* is the speed of sound in the medium. The transformation from Cartesian coordinates (*x*, *z*) to log-polar coordinates (θ,ρ) is given as

$$x=e^{\rho }\;\text{cos}\;\theta ,\;z=e^{\rho }\;\text{sin}\;\theta .$$ (2)

This coordinate transformation (Naghadeh and Riahi, 2013) is a conformal mapping that allows the wave equation to be expressed as

$$\frac{\partial^{2}p(\theta ,\rho ,t)}{\partial \theta^{2}}+\frac{\partial^{2}p(\theta ,\rho ,t)}{\partial \rho^{2}}=\frac{e^{2\rho }}{c^{2}}\frac{\partial^{2}p(\theta ,\rho ,t)}{\partial t^{2}}.$$ (3)

Taking the Fourier transform in *θ* and *t*, the wave equation can be written as

$$\frac{\partial^{2}\hat{p}(k_{\theta },\rho ,f)}{\partial \rho^{2}}=-{\left(2\pi \right)}^{2}\left({\left(\frac{fe^{\rho }}{c}\right)}^{2}-k_{\theta }^{2}\right)\hat{p}(k_{\theta },\rho ,f),$$ (4)

which has two solutions

$$\hat{p}(k_{\theta },\rho_{2},f)=H_{\pm }(k_{\theta },\rho_{1},\rho_{2},f)\hat{p}(k_{\theta },\rho_{1},f),$$ (5)

where $H_{\pm }(k_{\theta },\rho_{1},\rho_{2},f)$ is the propagator given by

$$H_{\pm }(k_{\theta },\rho_{1},\rho_{2},f)=\text{exp}\;\left(\pm j2\pi \int_{\rho_{1}}^{\rho_{2}}{\left({\left(\frac{fe^{\rho }}{c}\right)}^{2}-k_{\theta }^{2}\right)}^{1/2}d\rho \right)=\text{exp}\;(\pm j2\pi \left|k_{\theta }\right|[{\left({\left(\frac{fe^{\rho_{2}}}{ck_{\theta }}\right)}^{2}-1\right)}^{1/2}-\arctan\left({\left({\left(\frac{fe^{\rho_{2}}}{ck_{\theta }}\right)}^{2}-1\right)}^{1/2}\right)-{\left({\left(\frac{fe^{\rho_{1}}}{ck_{\theta }}\right)}^{2}-1\right)}^{1/2}+\;\arctan\left({\left({\left(\frac{fe^{\rho_{1}}}{ck_{\theta }}\right)}^{2}-1\right)}^{1/2}\right)]).$$ (6)

Since the integral inside expression (6) for the propagation filter has been evaluated, Eqs. (5) and (6) can be converted to a polar grid (θ,r), where $r=\sqrt{x^{2}+z^{2}}=e^{\rho }$. The propagation from the *r* = *r*_1_ manifold to the *r* = *r*_2_ manifold can be written as

$$\hat{p}(k_{\theta },r_{2},f)=H_{\pm }(k_{\theta },r_{1},r_{2},f)\hat{p}(k_{\theta },r_{1},f),$$ (7)

where the corresponding propagator $H_{\pm }(k_{\theta },r_{1},r_{2},f)$ is

$$H_{\pm }(k_{\theta },r_{1},r_{2},f)=\text{exp}\;(\pm j2\pi \left|k_{\theta }\right|[{\left({\left(\frac{fr_{2}}{ck_{\theta }}\right)}^{2}-1\right)}^{1/2}-\arctan\left({\left({\left(\frac{fr_{2}}{ck_{\theta }}\right)}^{2}-1\right)}^{1/2}\right)-{\left({\left(\frac{fr_{1}}{ck_{\theta }}\right)}^{2}-1\right)}^{1/2}+\arctan\left({\left({\left(\frac{fr_{1}}{ck_{\theta }}\right)}^{2}-1\right)}^{1/2}\right)]).$$ (8)

The angular spectrum method can then be written as

$$\begin{array}{c} p(\theta ,r_{2},f)=W(\theta )F_{k_{\theta }\mapsto \theta }^{-1}\{H_{\pm }(k_{\theta },r_{1},r_{2},f)F_{\theta \mapsto k_{\theta }}\{p(\theta ,r_{1},f)\}(k_{\theta },r_{1},f)\}(\theta ,r_{2},f), \end{array}$$ (9)

where W(θ) is a window function used to suppress the wraparound caused by the discrete Fourier transform. The forward and inverse Fourier transform operators $F_{\theta \mapsto k_{\theta }}$ and $F_{k_{\theta }\mapsto \theta }^{-1}$ in the angular dimension are

$$F_{\theta \mapsto k_{\theta }}\{f(\theta )\}(k_{\theta })=\int_{-\infty }^{\infty }f(\theta )e^{-j2\pi k_{\theta }\theta }d\theta ,$$ (10)

$$F_{k_{\theta }\mapsto \theta }^{-1}\{\hat{f}(k_{\theta })\}(\theta )=\int_{-\infty }^{\infty }\hat{f}(k_{\theta })e^{j2\pi k_{\theta }\theta }dk_{\theta }.$$ (11)

The polar form of the angular spectrum method presented here only accounts for a radially varying speed of sound, but the methodology could be modified into a Fourier split-step method (Schwab and Schmitz, 2018) that incorporates angular variations in the speed of sound in this polar coordinate system. This would require additional modeling of the angular variations in the speed of sound as phase screens at the end of each radial step of the polar form of the angular spectrum method. Although we apply a constant angular sampling of the pressure field at each radial step, zero-padding in the Fourier domain may be used to up-sample the wavefield in the angular dimension in a depth-dependent manner. Furthermore, this formulation can easily be extended to a three-dimensional (3D) geometry with a finite elevation (*y*-dimension) by using a cylindrical geometry.^1^

## 3. Validation of the angular spectrum method in polar coordinates

**Fig. 1.**
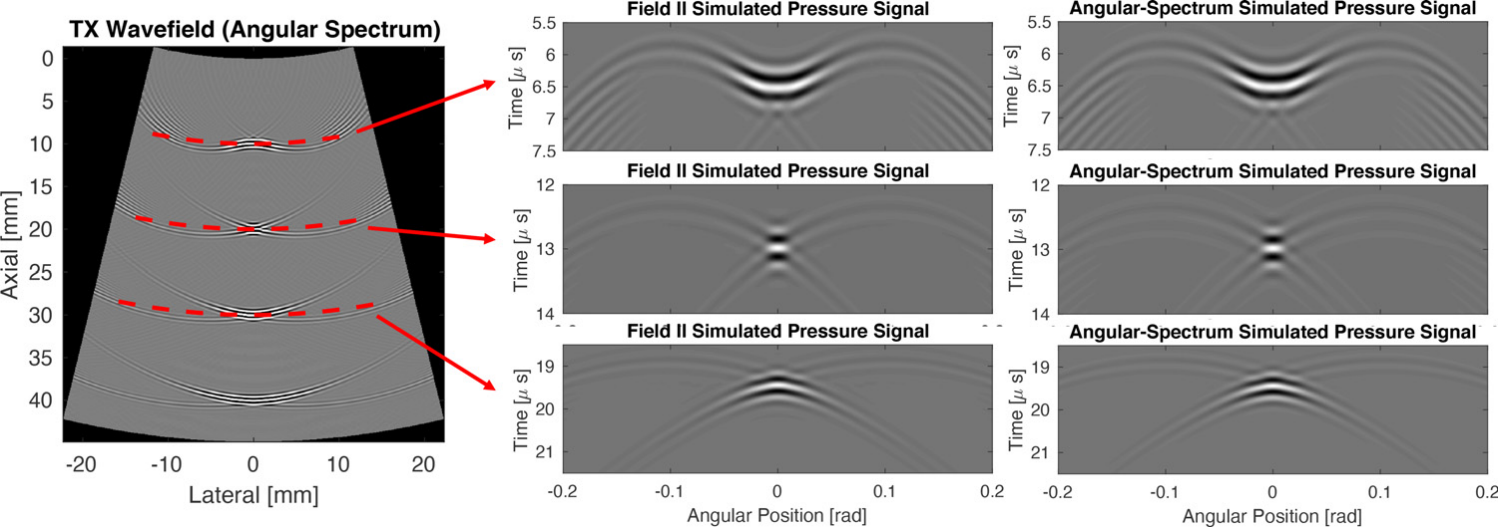
Comparison of the focused-transmit signal vs time at three imaging depths simulated in Field II and the angular spectrum method in polar coordinates. The pressure signal vs time is shown at three different cross-sections. The NRMSE between the two simulation methods is 3.7%.^1^

Field II (Jensen, 1996; Jensen and Svendsen, 1992) and the polar form of the angular spectrum method were used to calculate the wave-field from a focused transmit beam. Each simulation used a 128-element curvilinear probe with a 49.57 mm probe radius and 0.508 mm pitch. The pulses used in each simulation had a center frequency of 3.5 MHz at a fractional bandwidth of 50%. The transmit beam was focused at a depth of 20 mm and was produced by a 20-element subaperture. Each radial step of the polar angular spectrum method was 0.22 mm, which corresponds to half the wavelength for a 3.5 MHz transmit pulse in a 1540 m/s sound speed medium. Figure 1 shows the simulated wave-field versus time for the focused transmit beam using a Field II simulation and the proposed polar form of the angular spectrum method at three different depths in the medium. The normalized root mean square error (NRMSE) between the two simulation techniques is 3.7%. These results empirically demonstrate that the conformal mapping for the polar representation of the angular spectrum method is valid for the simulation of pressure fields from a curvilinear transducer array. This approach circumvents the need to convert wave-fields emitted by the curvilinear array to a Cartesian grid prior to the application of the angular spectrum method (Ilyina *et al.*, 2017). Last, although Field II yields a faster simulation of pressure fields by relying on analytical solutions for a constant sound speed in the medium, the angular spectrum method we present here could be extended to the frequency-domain simulation of pressure fields in a spatially varying sound speed medium. Next, we demonstrate the shot-profile migration imaging technique based on our polar angular spectrum method.

## 4. Application of the polar angular spectrum method to shot-profile migration

An image I(θ,r) can be formed by summing the time- or frequency-domain cross correlation of transmit $p_{tx(i)}(\theta ,r,t)$ and receive $p_{rx(i)}(\theta ,r,t)$ wave-fields for *N* transmit events,

$$I(\theta ,r)=\sum_{i=1}^{N}I_{i}(\theta ,r),$$ (12)

where

$$I_{i}(\theta ,r)=\int_{-\infty }^{\infty }p_{tx(i)}^{*}(\theta ,r,t)p_{rx(i)}(\theta ,r,t)dt=\int_{-\infty }^{\infty }p_{tx(i)}^{*}(\theta ,r,f)p_{rx(i)}(\theta ,r,f)df.$$ (13)

The transmit $p_{tx(i)}(\theta ,r,f)$ and receive $p_{rx(i)}(\theta ,r,f)$ wave-fields are calculated using the polar angular spectrum method,

$$p_{tx(i)}(\theta ,r+\Delta r,f)=W(\theta )F_{k_{\theta }\mapsto \theta }^{-1}\{H_{-}(k_{\theta },r,r+\Delta r,f)F_{\theta \mapsto k_{\theta }}\{p_{tx(i)}(\theta ,r,f)\}(k_{\theta },r,f)\},$$ (14)

$$p_{rx(i)}(\theta ,r+\Delta r,f)=W(\theta )F_{k_{\theta }\mapsto \theta }^{-1}\{H_{+}(k_{\theta },r,r+\Delta r,f)F_{\theta \mapsto k_{\theta }}\{p_{rx(i)}(\theta ,r,f)\}(k_{\theta },r,f)\},$$ (15)

where $p_{tx(i)}(\theta ,r=r_{0},f)$ and $p_{rx(i)}(\theta ,r=r_{0},f)$ represents the frequency spectra of the transmit pulse and receive channel data from each element on the array located at *r* = *r*_0_, the radius of the curvilinear transducer array. Knowledge of the transmit pulse spectrum *P*(*f*), transmit focal delays $\tau_{i}(\theta )$, and transmit apodization $A_{i}(\theta )$ for each transmit event *i* is used to form $p_{tx(i)}(\theta ,r=r_{0},f)$,

$$\begin{array}{c} p_{tx(i)}(\theta ,r=r_{0},f)=A_{i}(\theta )P(f)e^{-j2\pi f\tau_{i}(\theta )}. \end{array}$$ (16)

This image formation approach is referred to as shot-profile migration (Ali, 2021). Different receive apodizations may be applied to each transmit event by weighing the received channel data. However, the shot-profile migration framework prohibits the use of a location or depth-dependent receive apodization. Instead, an f-number dependent apodization may be applied by weighing the spatial frequency components of angular spectrum in the image formation process. Another important consideration when using the angular spectrum method for shot-profile migration is bandpass filter design. Throughout this work, we employ a flat passband that covers all the frequencies in the transmitted pulse. However, inclusion of all frequencies in broadband imaging cases can be computationally expensive. A hard truncation of frequencies can lead to axial ringing in the image, so tapering may be required to limit the image reconstruction to a narrower band of frequencies. However, this tapering of frequencies will reduce the axial resolution of the reconstructed image.

## 5. Using the polar angular spectrum method to demonstrate shot-profile migration in the time domain

**Fig. 2.**
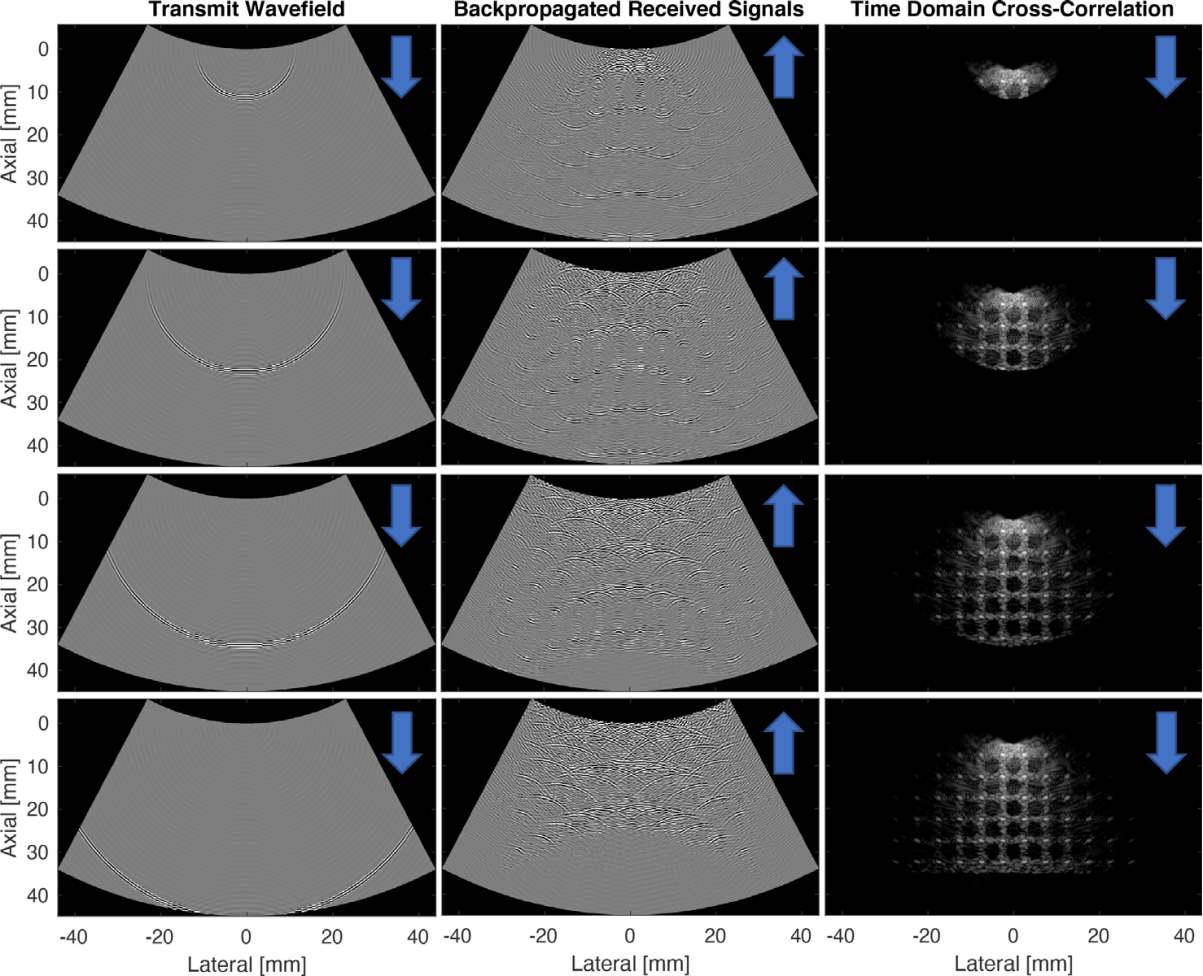
Image reconstruction by time-domain correlation of a single-element transmit wavefield and a backpropagated receive wavefield. Channel data were simulated in Field II.

Field II (Jensen, 1996; Jensen and Svendsen, 1992) was used to simulate received channel data from a single-element transmission in a diffuse-scattering medium with a grid of point targets and anechoic lesions. The simulation used a 128-element curvilinear probe with a 49.57 mm probe radius and 0.508 mm pitch. The transmit pulse had a center frequency of 3.5 MHz and a fractional bandwidth of 50%. The simulated receive channel data were used to demonstrate shot-profile migration in the time domain using the polar form of the angular spectrum method (Fig. 2).

Figure 2 uses the time-domain form of Eq. (13) to illustrate how the cross correlation of transmitted and receive wave-fields can be used to form an ultrasound image. As the transmitted waveform traverses over a region downwards into the medium, the back-propagated receive signals converge and focus at the locations where they were originally back-scattered. By correlating transmitted and back-scattered receive signals in this way, an image of back-scattered wave-field is formed. However, Fig. 2 only demonstrates this process using a single-element transmission. Commercial scanners typically use focused transmit beams rather than single-element transmissions. Section 6 demonstrates *in vivo* examples of frequency-domain shot-profile migration applied to channel data collected from focused transmissions on a commercial scanner.

## 6. Demonstration of Fourier-domain shot-profile migration using the polar angular spectrum method

A walking-aperture focused-transmit sequence implemented on a Siemens research scanner with an 5C1 probe was used to acquire radio frequency channel data from the abdomen of a healthy human volunteer under an IRB-approved protocol. Written informed consent was obtained. The Siemens 5C1 probe has 180 elements, a probe radius of 46.03 mm, and an element pitch of 0.3212 mm. Each transmit beam had a focal depth 97 mm. The walking aperture transmit sequence had a total of 121 beams ranging in angular span from −36.103º to +36.103º. These acquisitions are used to compare shot-profile migration using the polar form of the angular spectrum method to conventional delay-and-sum (DAS) beamforming for virtual-source synthetic aperture in focused transmit beams.

**Fig. 3.**
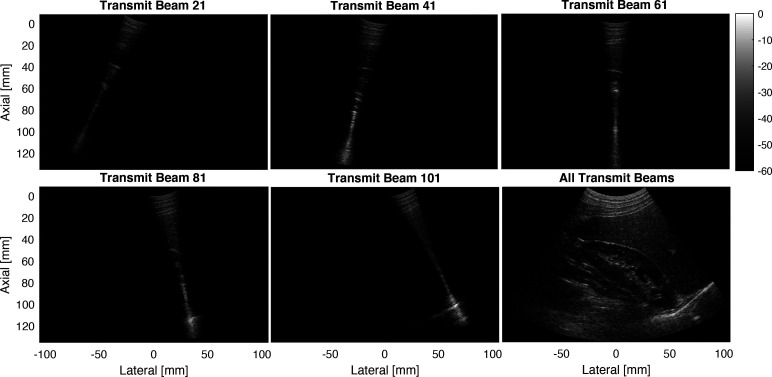
Shot-profile migration over focused transmit beams using a siemens 5C1 probe.

Figure 3 demonstrates synthetic aperture over focused transmit beams based on shot profile migration using channel data collected from the abdomen over the kidney. By accounting for focusing delays and the transmit apodization for each beam, shot-profile migration results in the optimal image for each transmit beam. The images from each transmit beam are coherently compounded to form the final synthetic aperture image. Shot-profile migration provides an alternative synthetic aperture imaging technique for focused transmit beams that does not require the virtual source model typically needed in DAS beamforming as previously shown in linear arrays (Ali, 2021).

**Fig. 4.**
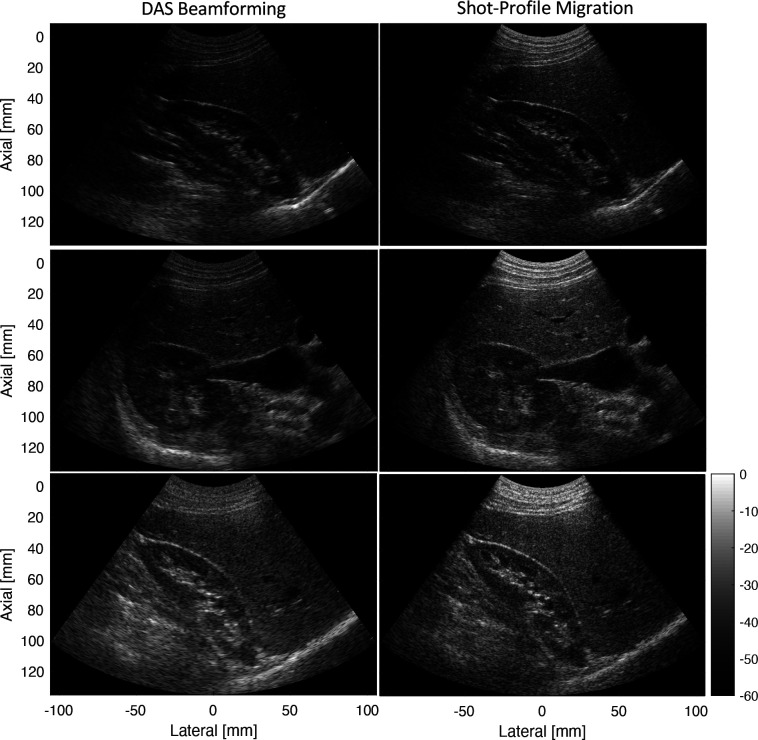
Comparison of shot-profile migration to DAS beamforming based on virtual-source synthetic aperture for a focused transmit sequence on a Siemens 5C1 probe.

Figure 4 compares shot-profile migration to DAS beamforming over several different views of the kidney. In each case, both shot-profile migration and DAS beamforming are used to perform synthetic aperture imaging across transmit beams. However, the DAS method relies on a virtual source model. As previously shown with linear arrays (Ali, 2021), shot profile migration results in greater imaging resolution than virtual source synthetic aperture because it better models the transmitted and received waveform without the drawbacks and limitations of assuming virtual sources. In each view of the kidney, the lateral resolution of the reconstructed images appears to improve as a result of applying shot-profile migration. A separate Field II simulation study of the transmit sequence used in these *in vivo* examples consistently shows at least a 35% improvement in point target resolution and a 7 dB improvement in anechoic lesion contrast. An additional *in vitro* example with a Verasonics C5–2v shows similar improvement in resolution and imaging contrast.^1^

However, the main drawback of the shot-profile migration approach is its computational expense. For a shot-profile migration image reconstruction that is $N_{\theta }$ points in the angular dimension and *N_r_* points in the radial dimension, the polar form of the angular spectrum method for shot-profile migration requires $O(N_{f}N_{r}N_{\theta }N\;\text{log}\;N_{\theta })$ operations, where *N_f_* is the number of frequency bins in *f* and *N* is the number of transmit events. Each radial step in *r* requires $N_{f}N_{\theta }N$ storage for the transmit and receive wave-fields. A virtual-source reconstruction based on DAS beamforming requires $O(N_{rx}N_{r}N_{\theta }N)$ operations, where *N_rx_* is the number of receive signals for each transmit event. However, DAS beamforming often requires the interpolation of time points from distant locations in memory, while the angular spectrum method is embarrassingly parallel across frequencies and transmit events. Although the radial propagation in the angular spectrum method is a serial process, the GPU can easily parallelize operations across transmit events and frequencies while performing the FFTs and inverse FFTs needed at each depth step very quickly.

As previously noted in the prior work on linear arrays (Ali, 2021), shot-profile migration also creates new opportunities for sound speed estimation. The migration velocity analysis techniques (Sava and Biondi, 2004) from seismic imaging were proposed for optimizing the shot-profile migration image with respect to the speed of sound to perform a tomographic reconstruction of the speed of sound. The technique presented in this paper would allow the robust implementation of such a technique in curvilinear arrays. Estimating and accounting for the spatially varying speed of sound in this way would also further enable source localization methods (Schoen, Jr. and Arvanitis, 2020) that previously relied on Cartesian forms of the angular spectrum method. This would enable the implementation of these sound speed estimation techniques in abdominal imaging, especially for the screening and detection of fatty liver disease (Imbault *et al.*, 2017).

## 7. Conclusions

This work demonstrates a polar form of the angular spectrum method that is best suited for applications related to curvilinear arrays. When compared to a Field II simulation of a focused transmit beam, the polar form of the angular spectrum method gives practically identical results (an NRMSE of 3.7% between simulation methods). This work also demonstrates that the polar form of the angular spectrum method can be used in shot-profile migration to reconstruct images with better lateral resolution than the virtual-source synthetic aperture imaging technique used in delay-and-sum beamformers, replicating results previously shown with linear arrays. In order to make our efforts more accessible to the broader research community, we have provided sample code and channel data online at https://github.com/rehmanali1994/CurvilinearAngularSpectrumMethod (DOI: 10.5281/zenodo.5822758).

## Footnotes

^1^
See supplementary material at https://www.scitation.org/doi/suppl/10.1121/10.0010536 for an alternative derivation of the polar form of the angular spectrum method and its extension to a 3D geometry, the Field II simulation study and *in vitro* example, and a better visualization of the error between the two simulation techniques.
